# Development of in vitro-grown spheroids as a 3D tumor model system for solid-state NMR spectroscopy

**DOI:** 10.1007/s10858-020-00328-8

**Published:** 2020-06-19

**Authors:** Reinier Damman, Alessandra Lucini Paioni, Katerina T. Xenaki, Irati Beltrán Hernández, Paul M. P. van Bergen en Henegouwen, Marc Baldus

**Affiliations:** 1 NMR Spectroscopy, Bijvoet Center for Biomolecular Research, Utrecht University, Padualaan 8, 3584 CH Utrecht, The Netherlands; 2 Cell Biology, Neurobiology and Biophysics, Department of Biology, Faculty of Science, Utrecht University, 3584 CH Utrecht, The Netherlands; 3 Pharmaceutics, Department of Pharmaceutical Sciences, Utrecht University, Universiteitsweg 99, 3584 CG Utrecht, The Netherlands

**Keywords:** Solid-state NMR, In-cell NMR, Spheroids, DNP, EGFR, Nanobodies

## Abstract

Recent advances in the field of in-cell NMR spectroscopy have made it possible to study proteins in the context of bacterial or mammalian cell extracts or even entire cells. As most mammalian cells are part of a multi-cellular complex, there is a need to develop novel NMR approaches enabling the study of proteins within the complexity of a 3D cellular environment. Here we investigate the use of the hanging drop method to grow spheroids which are homogenous in size and shape as a model system to study solid tumors using solid-state NMR (ssNMR) spectroscopy. We find that these spheroids are stable under magic-angle-spinning conditions and show a clear change in metabolic profile as compared to single cell preparations. Finally, we utilize dynamic nuclear polarization (DNP)-supported ssNMR measurements to show that low concentrations of labelled nanobodies targeting EGFR (7D12) can be detected inside the spheroids. These findings suggest that solid-state NMR can be used to directly examine proteins or other biomolecules in a 3D cellular microenvironment with potential applications in pharmacological research.

## Introduction

The contribution of cancer-derived cell lines to the understanding of molecular and cellular processes in the complex physiology of cancer has been immense. However, a full translation of the acquired knowledge towards patients can be challenging as the tumor microenvironment or tumor stroma is much more complex than an in vitro monolayer culture. Due to the globular shape of a tumor, concentration gradients exist for oxygen and nutrients, resulting in a different availability for the proliferative outside layer as compared to the core (Kim and Wu 2012). Moreover, as a result of the Warburg effect, synthesis of lactate, the end product of glycolysis, induces an acidification of the tumor and consequently results in altered gene expression and changes in the proteome. Eventually, as a result of these conditions, cells in the inner core die and form a necrotic core. Furthermore, the extracellular matrix (ECM) within the stroma forms a dense network of fibrils in which the tumor is embedded, driving tumor-specific intracellular signaling pathways ultimately leading to alterations in sensitivity of tumor cells to cancer therapies (Minchinton and Tannock 2006; Kim et al. 2008; Pylayeva et al. 2009; Liu and Agarwal 2010; Leight et al. 2012).

The intricate interplay between the microenvironment and tumor cells plays a vital role in the survival of the tumor, although the consequences of stromal conditions on protein functioning on a molecular level are poorly understood. In order to overcome these difficulties, a new dimension in cancer research has opened up by the advent of three-dimensional (3D) cell culture techniques. Several in vitro cell culture methods used in cancer research have been developed aiming at reproducing certain properties of the tumor and its microenvironment. Depending on the 3D cell culturing method followed, more complex aspects of cancer biology, like the effect of cellular architecture, cell–cell or cell-ECM (extracellular matrix) contact or tumor microenvironment, can be studied (Weigelt et al. 2014; Rijal and Li 2016; Ravi et al. 2017). Ultimately, patient-derived organoids are considered a highly complex system that offers the closest in vivo representation in vitro (Vlachogiannis et al. 2018), making them optimal platforms to be used in drug discovery. Overall, the microenvironment can have a prominent effect on protein function, supporting the necessity to study proteins within the most native context possible. Therefore, development of novel methods using 3D cell culture techniques for protein structural studies will contribute to a better understanding of the effect of the microenvironment on the structure–function relationship of proteins.

For a long time, solid‐state NMR (ssNMR) spectroscopy has been used to study complex molecules starting from applications on collagen (Torchia and Vanderhart 1976) or bone (Herzfeld et al. 1980), ranging to lipid bilayers (Seelig and Gally 1976) and membrane proteins (Kinsey et al. 1981). In recent years, progress in multidimensional and high-sensitivity ssNMR, i.e., Dynamic Nuclear Polarization [DNP, (Ni et al. 2013)] and fast magic-angle-spinning [MAS, (Andrew et al. 1958)] proton-detected ssNMR, in combination with advanced isotope labelling have further expanded the use of ssNMR experiments in native settings. For example bacterial cell preparations were used to examine β-barrel proteins (Renault et al. 2012a, b; Pinto et al. 2019), ion- and proton channels (Miao et al. 2012; Medeiros-Silva et al. 2016) or chaperones (Jacso et al. 2012; Baker et al. 2018; Pinto et al. 2019) in native membranes. In addition, retinal proteins (Ward et al. 2015; Yamamoto et al. 2015), electron transport proteins (Yamamoto et al. 2015), as well as globular proteins (Reckel et al. 2012; Schanda et al. 2014), peptides (Medeiros-Silva et al. 2018; Shukla et al. 2020) and entire protein complexes (Kaplan et al. 2015) were studied in the bacterial cell setting. Lastly, progress has also been made to produce isotope-labelled proteins in the context of ssNMR-based studies of human cells and their proteins (Kaplan et al. 2016a; Albert et al. 2018; Narasimhan et al. 2019; Luo et al. 2020). The latter advancements formed the conceptual basis for research described below.

Magnetic resonance has already been used to study tumors and shown the necessity of using 3D cell models by highlighting clear cancer-related metabolic changes (Bollard et al. 2002; Tee et al. 2018; Cox et al. 2019) including the use of MAS-based NMR (Bollard et al. 2002). In vitro-grown spheroids also show similar changes in their metabolism, indicating that this model system is a suitable mimetic of in vivo tumors. Moreover, metabolic biomarkers can now be readily used to identify tumors (Ramachandran et al. 2016; Ramachandran and Yeow 2017). Finally, Duer et al. have elegantly already demonstrated the potential of multidimensional ssNMR in cell tissue preparations and revealed 2D ssNMR correlations in murine ECM and tissue-culture derived ECM samples (Chow et al. 2014; Wong et al. 2015).

Here we aimed at developing a high-throughput approach to grow in vitro 3D tumor models, which are homogenous in size and shape by adopting the existing hanging drop method (Tung et al. 2011). In the current context, we used A431 cells that exhibit high expression levels of the EGF receptor [EGFR, (Haigler et al. 1978)]. Our data show that spheroids remain stable during extended ssNMR measurements and show distinct metabolic differences as compared to monolayer cell cultures. Moreover, our experiments reveal that isotope-enrichment of amino acids in spheroids is readily achieved using a standard culture medium. Finally, we adapted our earlier procedures (Beltrán Hernández et al. 2019) to study the [^13^C,^15^N]-labelled anti-EGFR nanobody 7D12 (Roovers et al. 2011) after penetration into the spheroids using sensitivity-enhanced DNP-ssNMR. Our data highlight the potential of conducting ssNMR-based studies on 3D cell cultures that in the future may reveal structure–function relationship of proteins in their native 3D cell microenvironment.

## Material and methods

### Cell culture

A431 (ATCC, CRL-1555, LGC Standards, Germany) were grown in Delbecco’s Modified Eagle’s Medium (DMEM: GIBCO, Invitrogen, Paisley UK) supplemented 10% Fetal Bovine Serum (FBS; Sigma F7524- Lot No BCBT6987), L-Glutamine (2 mM; Gibco #25030149) penicillin and streptomycin (Pen/Strep; Sigma #P0781) at 37 °C with an atmosphere containing 5% CO_2._

## Preparation of isotope-labelled spheroids

For [^13^C,^15^N] labelling of eukaryotic cells and medium preparation, we followed earlier reports (Kaplan et al. 2016a). In short, the labelled culturing medium was prepared by dissolving 1 g/L of [^13^C,^15^N] algal amino acid mixture (Cortecnet) into DMEM absent of unlabeled amino acids. The medium is further supplemented with dialyzed FCS and penicillin and streptomycin. A431 cells were grown for 2–3 passages in labelling medium to enrich cells in ^13^C,^15^N-labelled proteins. For the generation of spheroids, A431 cells were grown to a confluency of 70% in a monolayer culture after which they were dissociated of the culture dish by incubation with Trypsin/EDTA and resuspended in fresh labelling medium. The cell suspension was seeded into 384-well Perfecta3D hanging drop plates (MERCK, HDP1385) at a concentration of 50.000 cells/40 µL/well. Spheroids were grown for 48 to 76 h at 37 °C with an atmosphere containing 5% CO_2_ after seeding. The spheroids were harvested by centrifugation for 5 min at 300×*g*.

### Labelled nanobody production

Overnight cultures of *E. coli* BL21 codon + cells transformed with plasmid DNA encoding for 7D12-Myc-6xHis tagged behind under a T7 inducible promoter were grown in LB containing kanamycin. The overnight culture was diluted (1/100) into fully labelled M9 medium supplemented with 2 g/L ^13^C-D-glucose and 0.5 g/L ^15^NH_4_Cl. Bacteria were grown until an OD_600_ was reached after which protein production was induced by IPTG (0.5 mM) addition. 7D12 was produced overnight at 25 °C. The next day, bacteria were harvested by centrifugation for 20 min at 4000×*g* and resuspended in ice-cold PBS. Periplasmic preparation was harvested after at least 2 freeze–thaw cycles of the bacterial cell suspension followed by an additional centrifugation round of 30 min at 4000×*g*. His-tagged 7D12 was purified from the periplasm by immobilized metal-affinity chromatography (IMAC). The HisTrap HP Ni^2+^-Sepharose column was equilibrated with wash buffer (300 mM NaCl, 50 mM sodium phosphate, 10 mM imidazole, 2 mM β-mercaptoethanol). 7D12 was eluted from the column by the elution buffer (300 mM NaCl, 50 mM sodium phosphate, 100 mM imidazole, 2 mM β-mercaptoethanol). Protein fractions were pooled, and via buffer exchange, stored in PBS.

### Solid-state NMR MAS experiments

Conventional ssNMR were conducted using 3.2 mm triple-resonance (^1^H,^13^C,^15^N) magic-angle-spinning (MAS) eFree probe heads at static magnetic of 16.4T corresponding to 700 MHz resonance frequency (Bruker Biospin). Data were recorded at 273 K set temperature employing MAS rates of 5 kHz. Pulse schemes reflecting standard two-dimensional homonuclear ^13^C proton-driven spin diffusion [PDSD (Bloembergen 1949) with PARIS (Weingarth et al. 2009)] recoupling with 78 ms mixing time. Two-dimensional heteronuclear ^13^C^1^H J-based CH sequences were employed with INEPT (Morris and Freeman 1979) transfer steps to study mobile molecules. One-dimensional dipolar-based CH and NH cross-polarization [CP, (Pines et al. 1973)] sequences using 0.4 ms and 0.875 ms contact times respectively, and J-based CH sequence with INEPT transfer steps were additionally recorded. All experiments were recorded during SPINAL64 (Fung et al. 2000) decoupling during direct and indirect detection periods. Solution-state NMR HSQC spectrum (Bodenhausen and Ruben 1980) were recorded on a 900 MHz NMR instrument.

### Dynamic nuclear polarization: sample preparation

A431 spheroids were formed over a period of 76 h after seeding in unlabelled DMEM. The spheroids were harvested by centrifugation for 5 min at 300**×g** and washed with warm (37 °C) Leibovitz’s L-15 medium (ThermoFisher Scientific; Cat No 11415064).[^13^C,^15^N]-labelled 7D12 (500 nM) was added to the spheroids in Leibovitz medium and allowed to incubate with the spheroids for an hour. The spheroids were washed again to remove the excess of nanobody in medium. Then, unbound 7D12 was allowed to diffuse out of the spheroid during another incubation period in Leibovitz medium devoid of 7D12. Finally, the spheroids were washed, pelleted and resuspended in DNP juice (obtained by dissolving the DNP agent AMUPol (Sauvee et al. 2013) in 60% deuterated glycerol, 30% D_2_O and 10% HEPES, pH 7.4, for a final DNP radical concentration of 15 mM).

### Dynamic nuclear polarization: experiments

DNP measurements were performed at 100 K and 8 kHz MAS on a 3.2 mm triple-resonance (^1^H,^13^C,^15^N) magic-angle-spinning [MAS, (Andrew et al. 1958)] probe head at static a magnetic field of 9.4T corresponding to proton/electron resonance frequencies of 400 MHz/263 GHz (Bruker BioSpin) (Koers et al. 2014). All spectra were recorded at a MAS rate of 8 kHz using an 83 kHz SPINAL-64 proton decoupling. A recycle delay of 2 s was used for all experiments. To measure DNP enhancements, 0.2 and 0.6 ms contact times were used for H-C CP and H-N CP transfer, respectively. 100 Hz line-broadening were applied prior to Fourier transformation. DNP enhancements were obtained by scaling the signal intensities of spectra measured under DNP with the corresponding spectrum obtained without microwave irradiation at 100 K. The 2D DQSQ ^13^C, ^13^C correlation spectrum shown in Fig. 5 was obtained using the SPC5 (Hohwy et al. 1999) scheme an excitation and reconversion period of 2.5 ms for generation and subsequent reconversion of double quantum coherences. The 2D spectrum was processed using a 0.5π shifted sine squared window function in both dimensions.

### Micro-imaging experiments

For µMRI experiments, spheroids were grown for 76 h in normal DMEM after which they were harvested for sample preparation. The spheroids were washed once in ice-cold PBS and resuspended in Leibowitz 15 medium. For the MAS test, spheroids were spun down in a 3.2 mm rotor and immobilized using 2% agarose in Leibowitz medium. The 3.2 mm rotor was imaged inside of a 5 mm solution NMR tube.

The µMRI experiments were performed at a 22.2 T magnet corresponding to a resonance frequency of 950 MHz. For the micro-imaging experiments performed on immobilized spheroids, we used Diffusion Tensor Imaging (DTI) to generate contrast based on differences in the diffusion of water molecules in the sample (water-weighted contrast). The DTI standard protocol (Bruker Biospin, CA) was used to record the MR image of the spheroids inside the 5 mm solution NMR tube as well as inside the 3.2 mm rotor.

### Microscopy experiments

A431 spheroids were grown for 48–76 h at a cell density of 4.000 cells per well. The spheroids were harvested by centrifugation for 5 min at 300**×g** and fixed for 30 min at rt using 4% formaldehyde solution. The plasma membrane was permeabilized by incubation in 0.1% Triton X-100 in PBS for 15 min. Nuclei and F-actin were stained using 30 nM DAPI and phalloidin-A647 for 30 min at rt. The spheroids were washed 3 times using ice-cold PBS and mounted with Slowfade. Confocal images were obtained on a Zeiss LSM700 microscope using a 20 × Plan-Apochromat objective with a numerical aperture of 0.8.

### Nanobody binding assay

A431 cells were seeded into a 96-well nunclon culture plate (ThermoFisher) at a density of 10.000 cells/well on the day prior to the experiment. On the day of the experiment, cells were washed with binding buffer (DMEM, 1% BSA, 25 mM HEPES pH 7.4). A twofold serial dilution of [^13^C, ^15^N]-labelled 7D12, starting at a concentration of 613 nM, was prepared in binding buffer. The cells were washed and the serial dilution of the nanobody was added to the cells and allowed to incubate for 2 h at 4 °C. The cells were washed twice with ice-cold PBS before fixation using 4% formaldehyde for 15 min in binding buffer at rt and incubated in 1% BSA, PBS pH 7.4 for 1 h at rt. 7D12 was detected by incubating the cells for 1 h at rt with rabbit-α-VHH antibody in 1% BSA, PBS (Creative BioLabs). Goat-α-rabbit IRDye800 (Li-COR) was used to detect the primary antibody. Fluorescence intensity was measured using the Odyssey imaging system (Li-COR). Individual wells were quantified using ImageJ. Binding curve was created by one-site specific binding curve fitting using GraphPad Prism 5.

### Spheroid imaging

7D12 and R2 were expressed, purified by IMAC and conjugated to indicated dyes as described elsewhere (Oliveira et al. 2012; Beltrán Hernández et al. 2019). In short, his-tagged nanobodies were expressed in *E. coli* and purified from the periplasmic fraction by TALON purification and reconstituted in PBS. Alexafluor647-NHS (ThermoFisher Scientific) was conjugated to the nanobodies in PBS supplemented with 100 mM sodium bicarbonate, pH 8.3 for 1 h at rt. Unconjugated dye was removed by size exclusion chromatography using a Zeba desalting column (ThermoFisher Scientific). Binding of the nanobodies was verified using binding assays (Oliveira et al. 2012). Spheroids as described in ref. (Beltrán Hernández et al. 2019) were incubated at rt for 1 h with 25 mM of R2-A647 or 7D12-A647 after which the spheroids were extensively washed and fixed in 4% formaldehyde (Merck). Fixative was quenched using 100 mM glycine/PBS followed by membrane permeabilization using 0.5% Triton X-100 in PBS. Nuclei were stained by DAPI (Roche). Confocal imaging was performed on a confocal laser scanning microscope LSM700 (Carl Zeiss Microscopy GmbH) using a 40 × oil objective (EC Plan-NeoFluar, 1.3 NA). Images were analyzed in ImageJ**.**

## Results and discussion

**Fig. 1:**
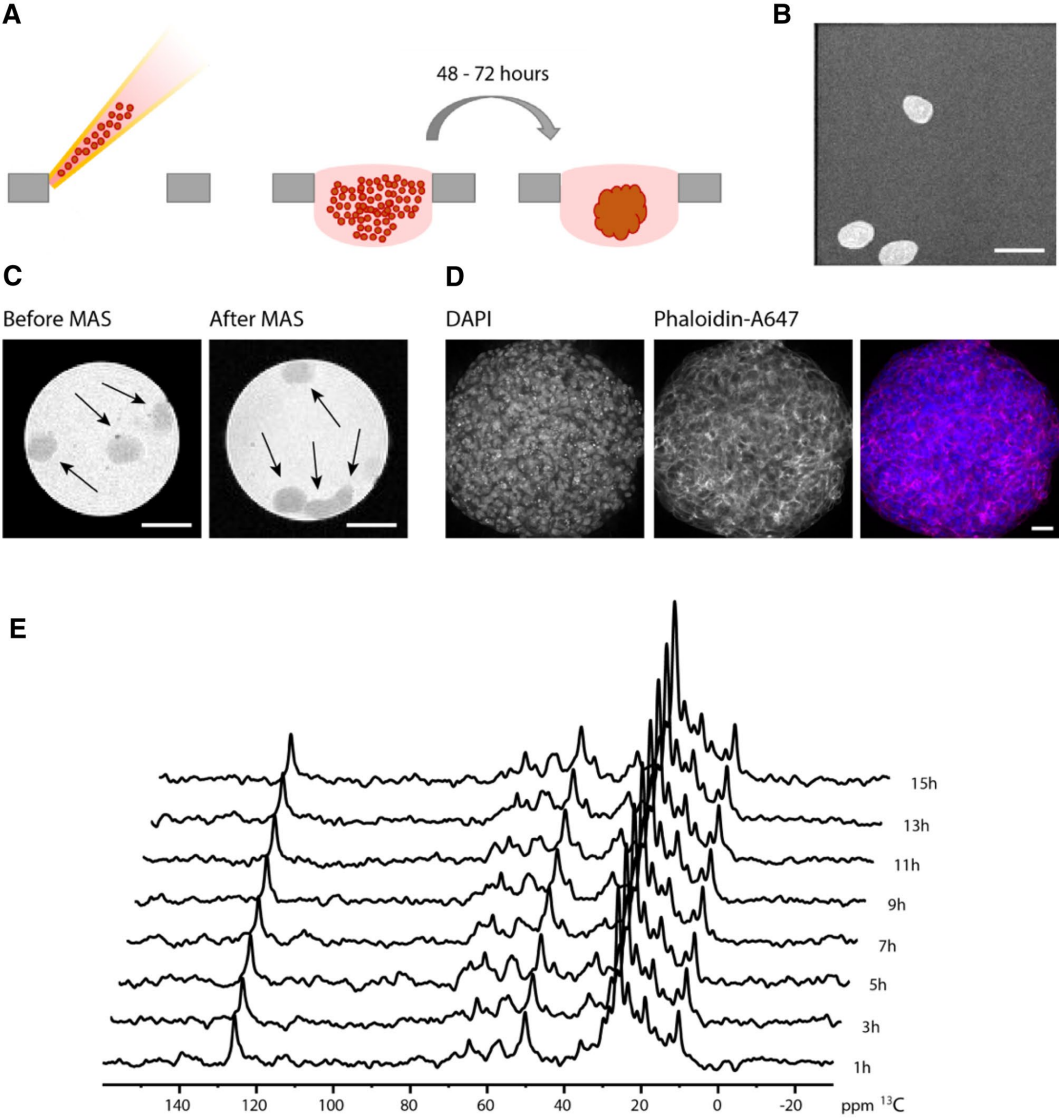
In vitro grown A431 spheroids are stable under ssNMR conditions. **a** Schematic representation of the hanging drop method. A431 cells are removed from the monolayer culture dish and seeded in a well plate suitable for hanging drop spheroid culturing. Homogeneous spheroids are formed between 48 and 72 h after seeding. **b** Side view of a micro-image of spheroids consisting of ~ 100.000 cells per spheroid. **c** A 3.2 mm rotor for ssNMR experiments was filled with spheroids consisting of ~ 100.000 cells per spheroid. The spheroids were imaged from the top prior to 5 kHz MAS and following MAS. Scale bars represent 1 mm. **d** Confocal imaging of spheroids stained for nuclei (DAPI, left) and actin (phaloidin-A647,middle) and merged images (right). Scale bar represents 40 µm. **e** Time course of 1D HC INEPT spectra recorded at a 2 h interval

A431 cells were grown in a monolayer culture until a confluency of 70–80% was reached. The cells were detached from the culture dish and transferred into a hanging drop 384-well plate (Fig. 1a). Within 24 h, several small spheroids were formed, which matured in the next 24–48 h to a single spheroid. To produce A431 spheroids for ssNMR experiments, spheroids were grown over a period of about 48 h until they reached a diameter of 600–700 µm (70–100.000 cells). They were harvested from the hanging drop plates by centrifugation and analyzed by confocal imaging. Nuclei (DNA) were stained using DAPI and actin filaments using phalloidin. This procedure confirmed that the spheroid contained tightly packed cells without the formation of cavities (Fig. 1b). Subsequently, spheroids were diluted in a 2% agarose solution and transferred in a 3.2 mm MAS rotor. Micro-imaging revealed that the spheroids were homogenous in size and shape (Fig. 1b). To test whether spheroids could sustain low speed magic angle spinning (MAS) during ssNMR experiments, spheroids were imaged after 5 kHz MAS for 1 h at ambient temperature. To perform MAS experiments we completely filled the rotor with spheroids. During MAS, the spheroids moved to the side of the rotor, and as a result, they showed some flattening of their shape into oval structures, but their integrity remained fully intact (Fig. 1c). 1D HC INEPT spectra were recorded on unlabeled spheroids at 2 h intervals to follow metabolic changes over time (Fig. 1e). No significant changes were observed during the recordings of the spectra, suggesting that the spheroids remained viable. Taken together, our data indicate that in vitro grown spheroids using the hanging drop method are homogenous and stable during ssNMR experiments.

**Fig. 2:**
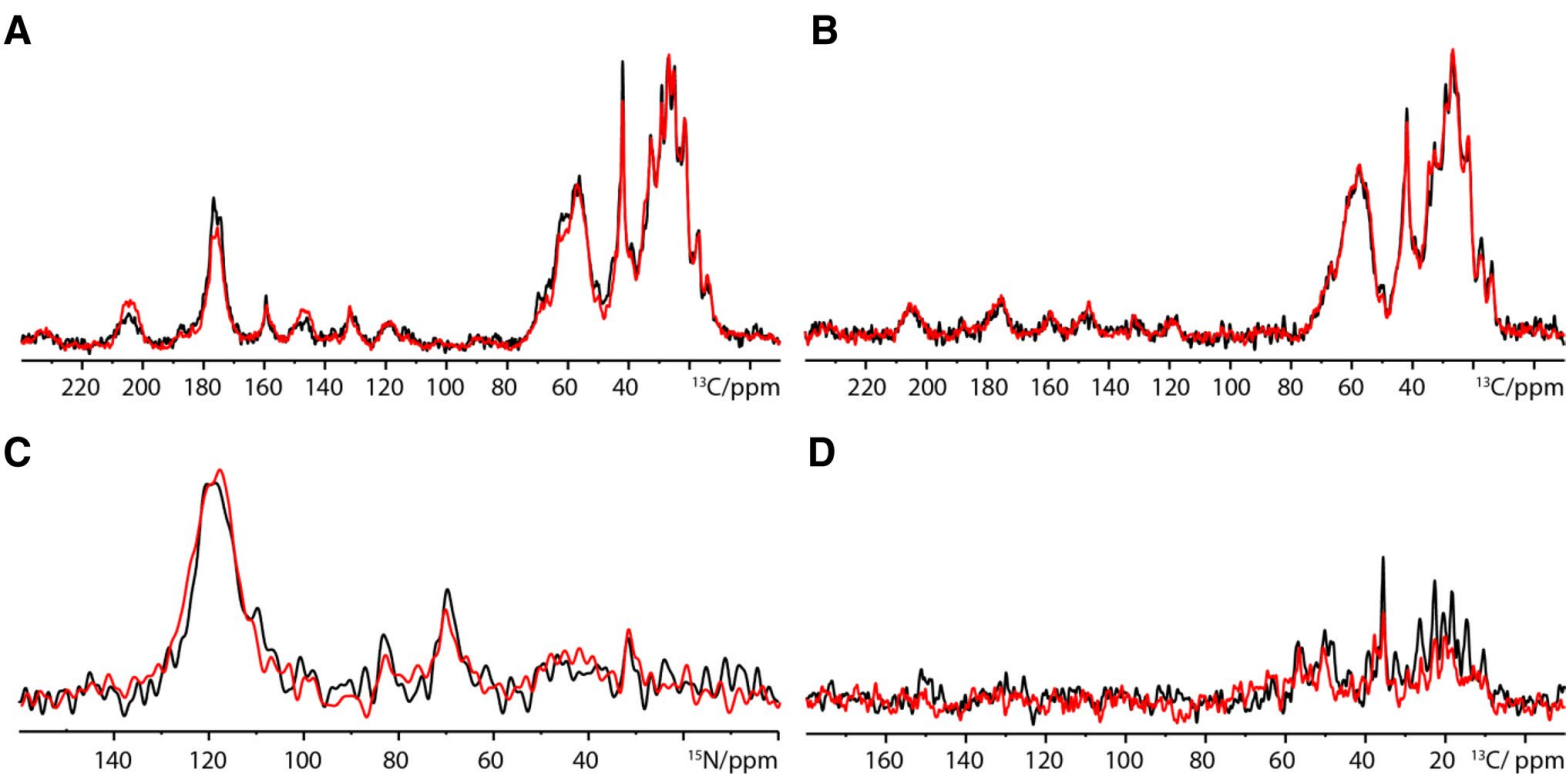
Comparison of 1D solid state NMR spectra on fully labelled A431 cells. 1D ssNMR data recorded on fully labelled A431 cells grown in a monolayer (black) or in a 3D spheroid (red) showing **a** ^13^C 90 degree spectrum, **b** ^1^H^13^C cross polarization, **c** ^1^H^15^N cross polarization and **d** ^1^H^13^C INEPT

For high-resolution and two-dimensional ssNMR experiments, incorporation of [^13^C,^15^N] isotope labels is required. Following earlier work (Kaplan et al. 2016a; Luo et al. 2020) we cultured A431 cells in a monolayer culture in DMEM medium containing [^13^C,^15^N]-labelled amino acids, before seeding them into hanging drop plates to form spheroids. A comparison of A431 cells grown in monolayer with cells grown in spheroids showed that the degree of labeling did not change upon the formation of spheroids and that the large majority of the rigidity-sensitive resonances originate from proteins (Fig. 2a, b). These data are in line with the nitrogen-detected cross polarization data showing clear backbone amide resonances and lysine and arginine side chain resonances (Fig. 2c). Interestingly, 1D HC INEPT spectra show a reduction in overall resonances observed in the spheroids sample when compared to the monolayer culture, suggesting that a spheroidal morphology affects the cells metabolites (Fig. 2d).

**Fig. 3:**
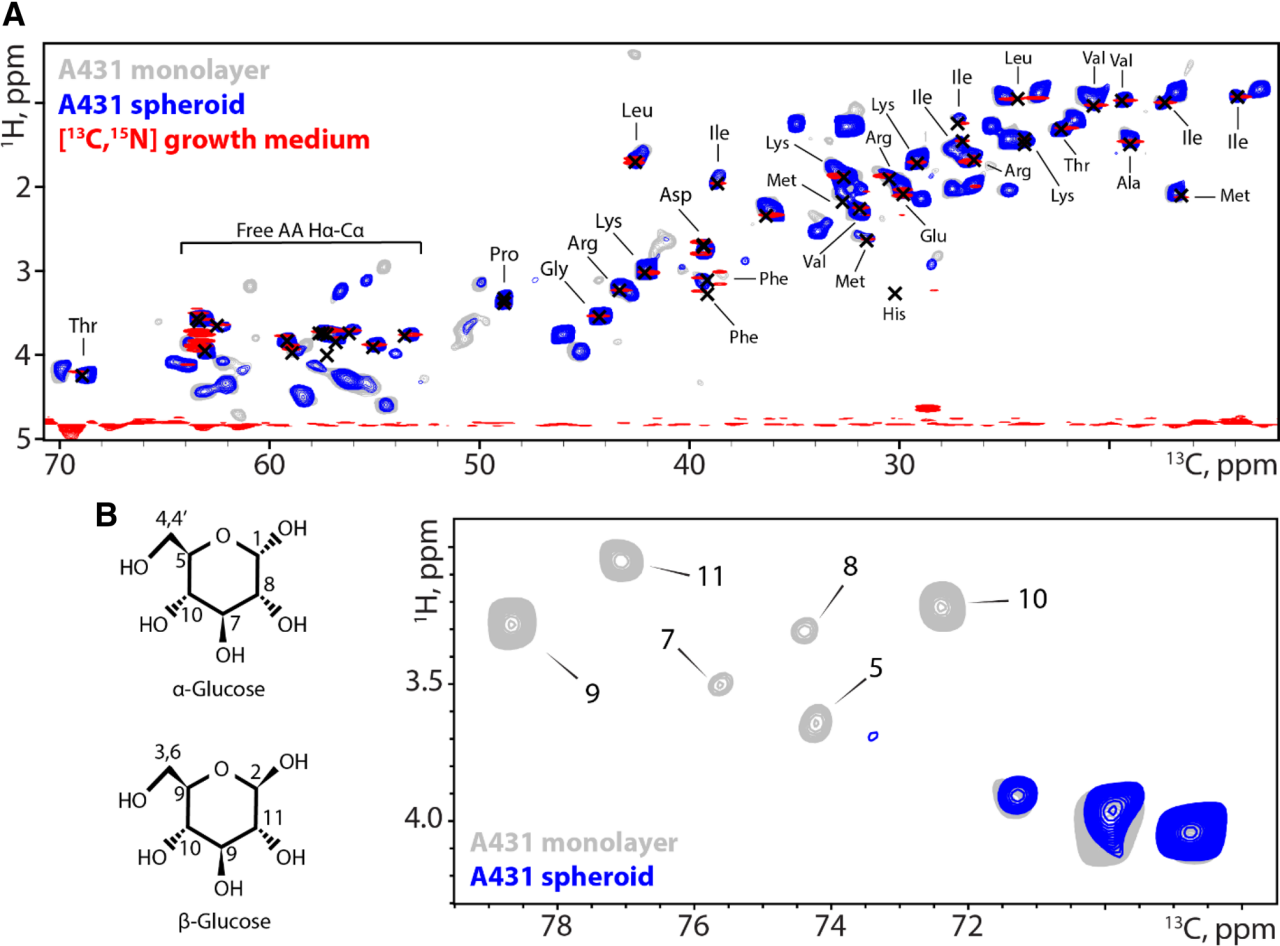
A431 spheroids incorporate spin labels from the culture medium and show metabolic alterations compared to monolayer cells. **a** HC INEPT spectrum recorded on labelled A431 cells from a monolayer culture and A431 spheroids at 5.000 kHz MAS. Red: culture medium; gray: cells grown in monolayer and blue: spheroids culture. Black crosses indicate solution state assignments as reported by the Human Metabolome Database. Note that glutamine, tryptophan and cysteine resonances are missing as they are added unlabeled. **b** Zoom in on the sugar region in the HC INEPT spectrum

To further investigate the effect of the spheroid culture on the metabolites inside the cell, two-dimensional HC INEPT spectra were recorded on monolayer- and spheroid cultures (Fig. 3a). The majority of the resonances observed are present in both spectra. When comparing the ssNMR-spectra to a solution NMR HC HSQC spectrum of labelled growth medium, we observe that many of these signals originate from the free amino acids in the growth medium. Based on these results it can be concluded that the isotopes in the growth medium are taken up and metabolically processed by the cells, mainly in protein synthesis. Interestingly, several peaks differ between the monolayer culture and the spheroid culture in either signal intensity or peak position, with certain correlations only appearing in one of the two cell culture condition. For example, the spectral region up field of free amino acids shows 6 resonances that can be assigned to a 6-ring sugar (Fig. 3b). These resonances are only present in the monolayer culture, while they are absent in the spheroid culture. Notably, it is well known that the availability of nutrients is much reduced in the spheroid culture, which results in the total consumption of the sugar source. We therefore concluded that the labelled amino acids available in the cell culture medium are consumed during proliferation and incorporated into several metabolic pathways, depending on their respective culture conditions.

**Fig. 4:**
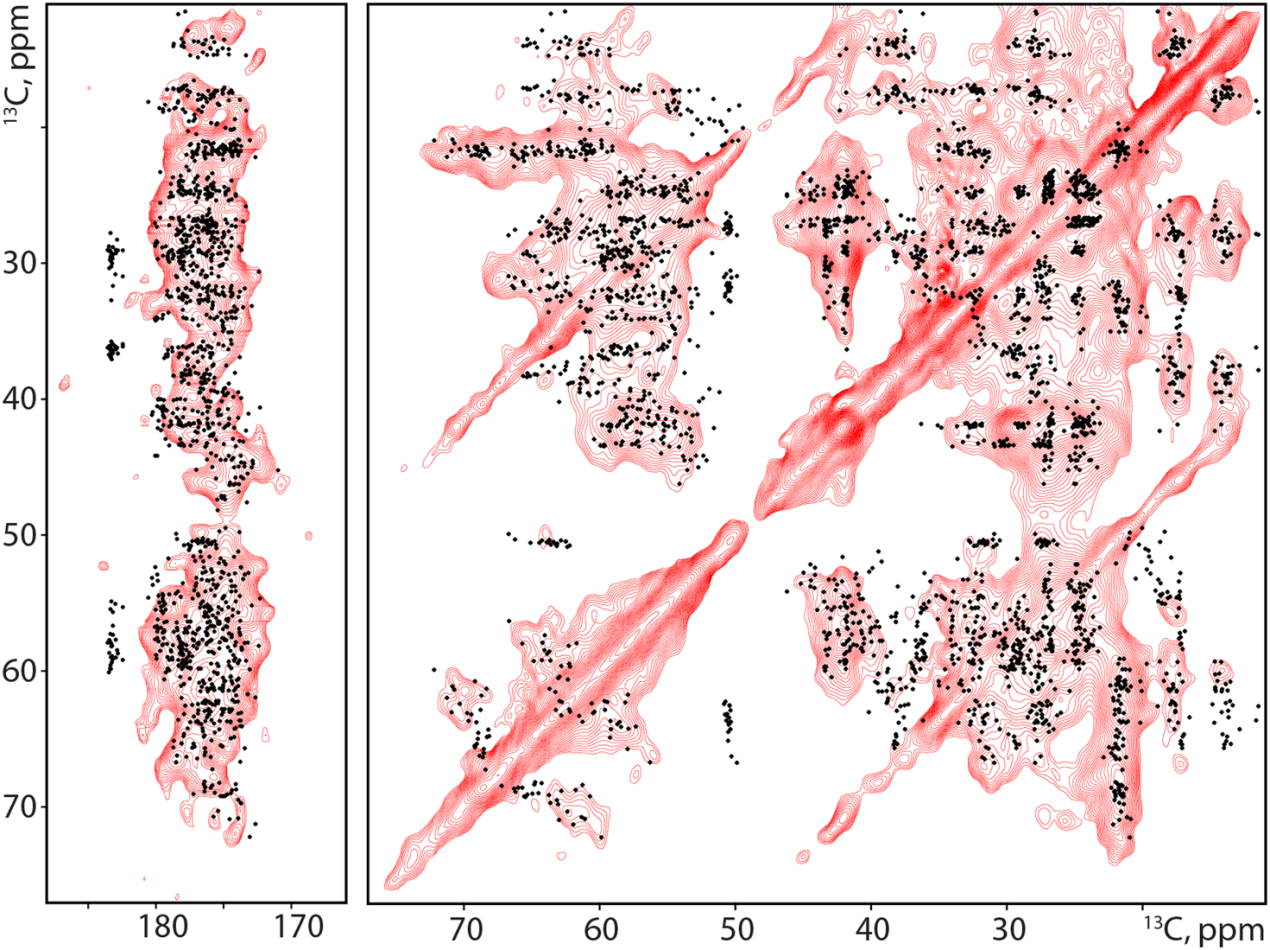
A431 spheroids are enriched in labelled proteins which remain folded under MAS conditions. Aliphatic region of a ^13^C-^13^C PARIS spectrum after a CP step showing protein signals. Actin is the most abundant protein in cellular ssNMR samples from A431 cells. Therefore, chemical shift predictions of actin (black dots) are overlaid on top of the spectrum, based on the F-actin structure model [PDB ID: 2ZWH(Oda et al. 2009)]. The predictions are in qualitative agreement with the observed resonances

As seen before, dipolar-based carbon-detected ssNMR spectra of fully labelled cells are dominated by protein resonances (Kaplan et al. 2016a). 2D CC PARIS spectra recorded on A431 spheroids confirmed the high degree of proteinaceous signals in the aliphatic region of the spectrum (Fig. 4). For certain amino acid types, e.g. serine and threonine, (Cα-Cβ and Cβ-Cγ) cross peaks were observed, suggesting the presence of different secondary structure elements. This observation would be in line with the notion that proteins inside the cells remain folded. Based on our previous experiments, actin is one of the most abundant proteins inside A431 cells (Kaplan et al. 2016a). Hence, we used FANDAS (Gradmann et al. 2012; Narasimhan et al. 2018) to predict chemical shift correlations using the structural model of actin (Oda et al. 2009). The resulting correlation pattern is in qualitative agreement with the data recorded (Fig. 4, black dots), indicating that the CC PARIS spectra are dominated by rigid proteins, likely including actin. It is important to note that, due to the general labeling scheme applied here, all proteins or other amino acid-derived metabolites are observed. This makes it difficult to study the single structures without further preparative steps such as described before (Kaplan et al. 2016a; Narasimhan et al. 2019).

**Fig. 5:**
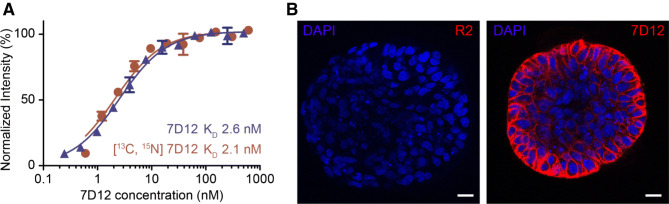
Binding of nanobody 7D12 to EGFR. **a** Binding assay of non-labeled 7D12 (blue) and [^13^C, ^15^N]-labelled 7D12 (red) to A431 cells. **b** Confocal microscopy images of A431-derived spheroids after 1 h at rt of incubation with either nanobody R2-A647 (red; left panel) or 7D12-A647 (red; right panel). Nuclei are stained by DAPI (blue). Scale bar represents 20 µm

In order to study single proteins inside 3D cellular structures, we resorted to a different approach, where we added labelled protein to unlabeled spheroids. Previous work performed on the nanobody 7D12 has shown that it binds with high affinity to domain III of the ectodomain of EGFR (Roovers et al. 2011; Schmitz et al. 2013). Incorporation of the spin labels into 7D12 did not affect binding to EGFR on A431 cells (K_D_ = 2.1 nM) (Fig. 5a). To ensure 7D12 penetration into the spheroid and subsequent binding to EGFR, we performed confocal microscopy experiments. As a negative control we imaged penetration and binding of R2 (Beltrán Hernández et al. 2019), a non-specific nanobody. R2 and 7D12 were directly conjugated to fluorophore Alexa647 and allowed to penetrate into the spheroid for 1 h after which spheroids were extensively washed with binding buffer. R2 is efficiently removed from the spheroids while binding of 7D12 is detected in the outer layer of the spheroids (Fig. 5b). These data demonstrate the presence of bound 7D12 inside the spheroid.

**Fig. 6:**
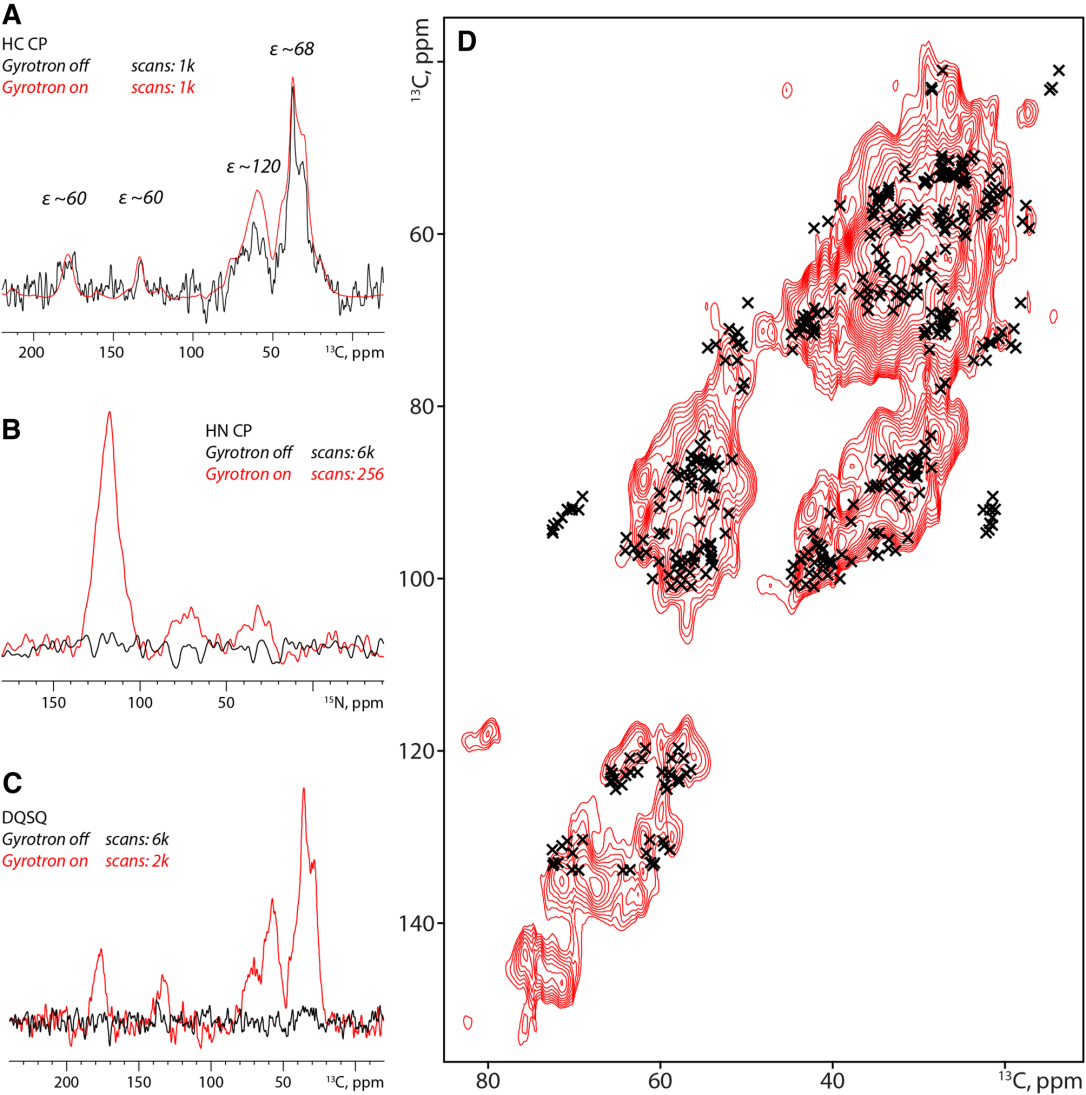
DNP-supported ssNMR on labelled nanobodies inside spheroids. **a**, **b** DNP-supported 1D HC (**a**) and HN (**b**) cross-polarization ssNMR experiments highlight the presence of labelled 7D12 inside A431 spheroids. The overall enhancement (ε) recorded on protein signal is ~ 80 while on the glycerol is approximately 120. **c** 1D DQSQ-filtered ^13^C,^13^C ssNMR spectra confirm the presence of labelled 7D12 inside the sample. **d** 2D DQSQ spectrum recorded on 7D12-doped A431 spheroids highlighting the conserved folding of 7D12 inside spheroids. Black crosses represent chemical shift predictions of 7D12 based on its crystal structure, complexed to EGFR Domain III of the ECD (PDBid 4KRL)

To study such preparations by ssNMR, we added [^13^C,^15^N]-labelled 7D12 to spheroids that were grown under normal culture conditions and allowed the nanobodies to penetrate into the spheroid for 1 h in Leibovitz’s L-15 medium. Subsequently, unbound 7D12 was removed by extensive washing with fresh Leibovitz’s L-15 medium. We estimate the total amount of 7D12 inside the spheroid to be 50% of the amount of EGFR expressed in the spheroid (i.e., ~ 10^11^ 7D12 nanobodies), making the use of conventional carbon-detected ssNMR challenging. Hence, to improve the signal-to-noise ratio, we resorted dynamic nuclear polarization (DNP) where NMR signals are greatly enhanced by polarization transfer from electrons. We observed a protein enhancement of ~ 80 (Fig. 6a) at 400 MHz under DNP conditions which is at least comparable to values seen in other complex biomolecules (Koers et al. 2014; Kaplan et al. 2016b). Moreover, large enhancements were also observed for nitrogen-detected cross-polarization experiments, where clear backbone amide and side-chain nitrogen resonances are observed (Fig. 6b). To ensure the NMR signal originates exclusively from 7D12 and not from the ^13^C or ^15^N natural abundance of the sample, we recorded 1D and 2D double quantum single quantum (DQSQ) experiments. These data show a clear protein signal and no other metabolites (e.g. lipids or sugars), which suggests that we are able to detect labeled 7D12 that is, according to our results presented in Fig. 5, mostly bound inside the spheroids (Fig. 6c). Moreover, 2D DQSQ spectra reveal a close correlation with chemical-shift predictions based on the crystal structure of 7D12 bound to DIII of EGFR (Schmitz et al. 2013) (PDB: 4KRL), confirming the presence of well folded and (see Fig. 5a**)** functional 7D12 (Fig. 6d). Note that the additional signals observed around 70–78 ppm originate from natural abundance glycerol, which is added to the sample to prevent disruption of the spheroids. Taken together, our data show that for, NMR standards, comparatively small amounts of labelled proteins can be added to a complex spheroid environment and can be studied using sensitivity-enhanced ssNMR.

## Conclusions

3D cell culture techniques such as spheroids and organoids are increasingly recognized as improved in vitro systems to study cells within their cellular context. This includes the effects of cellular contacts with other cells, the extracellular matrix within tissues and specific growth conditions, such as low pH, which is particularly characteristic for the tumor microenvironment. The enhanced complexity plays an important role in the translation of in vitro data towards in vivo situations, as the heterogeneity in the sample may influence the resulting structure–function relationship of proteins or other (bio)molecules.

Currently, efforts are made to obtain a better understanding of the effect of the native environment on protein structure and functioning. However, such experiments are often performed on bacterial- or mammalian-derived membranes for membrane proteins, or they involve in-cell NMR studies inside a single cell for cytoplasmic proteins. The additional complexity introduced by 3D cell culture techniques e.g. ECM, nutrient gradients, cell–cell contacts and subsequent changes in the metabolome could potentially have a strong influence on the protein structure–function relationship (Siegal and Selenko 2019). To obtain knowledge on these aspects, ssNMR can contribute by studying protein structure and dynamics at atomic resolution without the limitation of sample size. Here, we have presented a method to grow simplified tumor model systems in vitro that are stable during extended ssNMR measurements. Our results show that the hanging drop method for the generation of spheroids can be scaled-up to obtain large numbers (150–200) of homogenous spheroids, which can be readily manipulated. Importantly, the spheroids are stable and remain intact during MAS for several days. Secondly, we observe clear differences in the metabolic profile of cells grown in a monolayer culture or 3D culture, emphasizing the necessity to explore 3D systems for structural biology. Thirdly, spin labels can easily be introduced into growing spheroids during culture and used to record 2D correlation spectra on proteins or other cellular components Finally, other labelled biomolecules such as protein ligands, as shown in this work, or additional components of the ECM can be added. These findings open up novel approaches for studying proteins or other biomolecules at atomic resolution in a complex cell microenvironment.

Even though the spheroids presented here consist of a single cell type, the hanging drop method can be easily manipulated to include other factors that contribute to a tumor microenvironment. For example, several types of cells are typically found within a tumor e.g. fibroblasts and immune cells and could potentially be included in the hanging drop method by adapting co-culture protocols. Moreover, adding ECM fibrils such as collagen to the hanging drop, will result in their incorporation by the growing spheroids to form an integrated network of fibril-cell and cell–cell contacts. Additionally, advances in the delivery of labelled proteins into mammalian cells (Narasimhan et al. 2019) allow for new developments in sample preparation for sensitivity-enhanced ssNMR at higher magnetic fields (Koers et al. 2014), enabling the study of individual proteins inside 3D cell cultures at atomic resolution. We envision that such experiments will provide important insights into the role of the microenvironment on the structure–function relationship of proteins and contribute to pharmacological research, thereby bridging the gap between structural and cell biology.
